# Discovery of Aphid Lethal Paralysis Virus in *Vespa velutina* and *Apis cerana* in China

**DOI:** 10.3390/insects10060157

**Published:** 2019-06-03

**Authors:** Dahe Yang, Hongxia Zhao, Junming Shi, Xiang Xu, Yanyan Wu, Rui Guo, Dafu Chen, Xinling Wang, Shuai Deng, Sa Yang, Qingyun Diao, Chunsheng Hou

**Affiliations:** 1 Institute of Apicultural Research, Chinese Academy of Agricultural Sciences, Beijing 100093, China; yangdahe@126.com (D.Y.); xuxiang@caas.cn (X.X.); wuyanyan@caas.cn (Y.W.); wangxinlingjiayou@126.com (X.W.); Shdeng11@163.com (S.D.); Sayang1994@163.com (S.Y.); 2 Key Laboratory of Pollinating Insect Biology, Ministry of Agriculture, Beijing 100093, China; 3 Graduate School of Chinese Academy of Agricultural Sciences, Beijing 100081, China; 4 Guangdong Key Laboratory of Animal Conservation and Resource Utilization, Guangdong Public Laboratory of Wild Animal Conservation and Utilization, Guangdong Institute of Applied Biological Resources, Guangzhou 510260, China; hxzh110@126.com; 5 State Key Laboratory of Virology, Wuhan Institute of Virology, Chinese Academy of Sciences, Wuhan 430071, China; Sjm19901002@sina.com; 6 College of Bee Science, Fujian Agriculture and Forestry University, Fuzhou 350002, China; ruiguo@fafu.edu.cn (R.G.); dfchen826@fafu.edu.cn (D.C.)

**Keywords:** *Vespa velutina*, *Apis cerana*, aphid lethal paralysis virus, virus vector, native pollinator

## Abstract

Honey bees are essential to the functioning of terrestrial ecosystems. However, despite no single factor being blamed for losses of honey bee colonies in Europe and the USA, viruses have been considered as a major driver. Moreover, a virus vector can enhance the titer and virulence of virus such as *Varroa destructor* can change the virulence of the deformed wing virus. Here, we report molecular evidence for aphid lethal paralysis virus (ALPV) infecting *Vespa velutina*, which is an important predator of honey bees, especially of *Apis cerana*. Viral replication and phylogenetic analysis indicated that ALPV can not only replicate in *V. velutina* and *A. cerana*, but ALPV from *A. cerana* (ALPV-Ac) was also significantly associated with that of *V. velutina* (ALPV-Vv), though distinct from those of *Apis mellifera* (ALPV-Am). The host state posterior probability displayed that *V. velutina* is the main viral reservoir between *V. velutina* and *A. cerana*. Our results show ALPV had expanded host diversity resulting in potential impacts on the health of pollinators, even on the pollination ecosystem. We suggest further studies should investigate potential risks and impacts on pollinator populations of hornets. These results should have an impact conservation efforts focused on sustaining native pollinator abundance and diversity, and therefore, the crucial ecosystem services that they provide.

## 1. Introduction

Although honey bees (*Apis mellifera*) play a vital role in plant diversity and agricultural ecosystems, they suffer from a variety of pathogens, including viruses, bacteria, fungi, and parasites, leading to a large loss in bee populations in the USA and Europe in the past few decades [1], but little impact on Chinese honey bee colonies [2,3]. Viruses are considered as the major factor associated with significant loss of colonies, especially overwintering honey bee colonies, and include deformed wing virus (DWV) and Israeli acute paralysis virus (IAPV) [4]. These viruses usually produce covert infections and do not induce visible damage if honey bees do not encounter other stressors [5]. Nevertheless, as pathogen vectors, predators play a critical role in the outbreak and transmission of pathogens among honey bees or other pollinators due to shared common plant resources, despite there being increasing reports about virus breakouts via other means such as pesticides [5,6,7]. 

However, one of the primary predators of honey bees, hornets (*Vespa* spp.), might also be the other main or occasional hosts of viruses. One species, *V. velutina nigrithorax*, is drawing great concern from public authorities and beekeepers due to its rapid spread in Europe and South Korea, causing bee populations to decline [8,9]. Hornets not only threaten honey bee population growth, but also interact with honey bees or wild pollinators such as the wild Chinese honey bee (*Apis cerana*) and can act as viral reservoirs to infect them through predation [6,10]. The hornet, especially the yellow-legged Asian hornet *V. velutina*, has been considered an important invasive species in Europe [11]. Recently, it was found that *V. velutina* in Belgium was infected by the Moku virus, leading to its potential spread to honey bees [10]. Both aphids and *V. velutina* share a number of plant food resources like sugary fluid [12,13]; therefore, a potential interaction between aphids and *V. velutina* can lead to the transmission of viruses. In addition, it was reported that aphid lethal paralysis virus (ALPV) has been detected in Spanish honey bees [14]. To explore whether ALPV, which is closely related to the *Dicistroviridae* virus, a common family of insect viruses, is present in Chinese *V. velutina* and honey bees, we screened for its presence in *V. velutina* and *A. cerana* samples from five provinces in China, and we analyzed its replication ability in *V. velutina* and *A. cerana* and its phylogenetic relations with other known ALPVs.

## 2. Materials and Methods

From field observations, honey bees were frequently attacked by hornets (Figure S1A). We collected *V. velutina nigrithorax* at the entrance of bee hives as well as *A. cerana* samples from Shannxi, Beijing, Jiangxi, Qinghai, and Guangdong provinces between July 2016 and December 2017. In brief, around 5–10 *V. velutina* workers and 50 bees were randomly sampled at the each hive entrance and inside the colonies, and RT-PCR was performed on pooled samples for detecting the presence of ALPV following the manufacturer’s protocol with a pair detection primers [13]. In addition, we also collected *Apis mellifera* samples (pooled samples of ~50 bees) from which we collected *V. velutina* and *A. cerana*, but we did not find ALPV. Both *V. velutina* and honey bee samples were homogenized using a Tissueprep® homogenizer (Getting Scientific Instrument Ltd., Beijing, China) in a 15 mL sterile tube, using the RNA Purification Kit (Promega Corp., Madison, WI, USA) for RNA extraction, according to the manufacturer’s instructions. cDNA was used to amplify the full-length genome of ALPV with the designed primers when a sample was detected positive for ALPV (Table S1). 

For detecting the presence of ALPV, PCR amplifications were carried out in a volume of 20 μL. This PCR mixture contained template cDNA, 2× GoTaq Mix (Promega, Madison, WI, USA), and primers as described in Table S1. Amplification was run on an ABI PCR machine (Gene Company Ltd., HongKong, China) for 3 min at 94 °C; followed by 32 successive cycles of 30 s at 94 °C, 30 s at 55 °C, and 72 °C for 1 min; and a final extension of 10 min at 72 °C. The PCR products were examined by electrophoresis on a 1.2% agarose gel containing Gold View II nucleic acid stain (SBS Genetech Corp. Ltd., Beijing, China). For determining the copy numbers of the ALPV in *V. velutina* and *A. cerana,* quantitative estimation of positive and negative-sense RNA strands was performed by using the tagged primers as described in Table S1. Housekeeping gene primers used were β-actin forward 5′-TTGTATGCCAACACTGTCCTTT-3′ and β-actin reverse 5′-TGGCGCGATGATCTTAATTT-3′, respectively [15]. Quantitative PCR was performed using the SYBR-Green-based KapaSybrR Fast qPCR kit (Sigma-Aldrich, St. Louis, MO, USA) according to the manufacturer’s instructions. The cycling protocol was 95 °C for 10 min followed by 40 cycles of 95 °C for 10 s, 60 °C for 15 s, and 72 °C for 20 s. Standard curves were prepared by performing real-time qPCR with serial 10-fold dilutions of known concentrations of the ALPV-specific amplicons. The results were analyzed using 9600 Plus Software.

Target fragments were sequenced and assembled using Vector NTI Advance 11 software. Besides the current obtained sequences of *V. velutina* and *A. cerana* from China, the ALPV genomic sequences deposited in GenBank were used for the dataset construction, including the corresponding information of collection dates and locations. In total, 27 sequences from nine different countries were used. We constructed the phylogenetic analysis based on the full-length nucleotide sequences of RNA dependent RNA polymerase (RdRp). The sequences of RdRp genes of ALPVs were used to perform bioinformatics analysis due to its being conserved in discistrovirus [16]. CLUSTAL W was used for multiple sequence alignments, and phylogenetic analysis was carried out on each dataset via the BEAST v1.10.2 package [17]. 

The gamma distribution (GTR+I+G) model was identified using jModelTest as the best-fit model for phylogenetic analyses with both two datasets [18]. The Bayesian Markov chain Monte Carlo (MCMC) method, available in the BEAST v1.10.2 package [17], was used to estimate divergence times, substitution rate, and perform the Bayesian analysis using the GTR+I+G model of nucleotide substitution, a strict clock model and coalescent constant population tree prior. For each dataset, BEAST analysis was run for 40 million generations to achieve a convergence of parameters by calculating the effective sample size (ESS > 200) using TRACER v1.7.1 (http://beast.community/). The maximum clade credibility (MCC) tree was computed using the TreeAnnotator program within the BEAST package, with the first 10% of trees removed as burn-in, and visualized by Figtree v1.4.3. Bayes factors were calculated to evaluate the transmission support, using a Bayes factor (BF) of >3 as a cut-off.

## 3. Results

As *V. velutina* is one of major predators of *A. cerana* in China, we collected *V. velutina* at the entrance of bee hives as well as *A. cerana* samples, and we found ALPV in *V. velutina* and *A. cerana* (Figure S1B). Although no clinical signs were found in *V. velutina* and *A. cerana,* the detection of ALPV negative-sense RNA strands strongly suggested that ALPV can replicate in them (Figure S1C). Moreover, the most conserved motif, YGDD, was identified in ALPVs and other members of the *Dicistroviridae* family, including three honey bee viruses and cricket paralysis virus (CrPV) (Figure S1D). Additionally, we also screened *A. mellifera* samples from locations where we collected *V. velutina* and *A. cerana*, but we did not find ALPV. 

**Figure 1:**
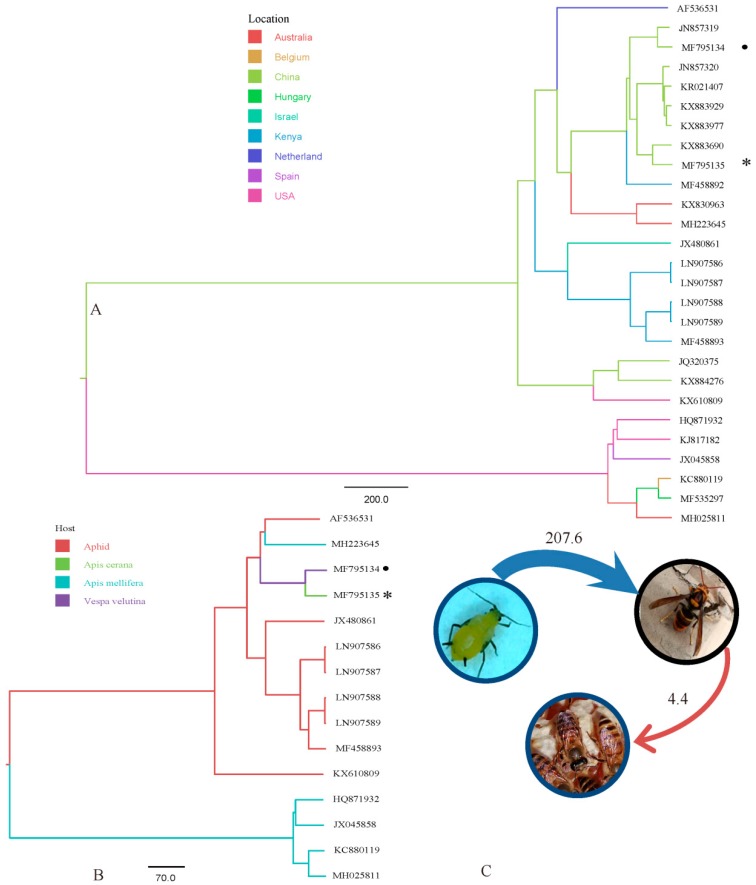
Maximum clade credibility (MCC) phylogeny for RNA dependent RNA polymerase (RdRp) gene of aphid lethal paralysis virus (ALPV) showing inferred host states. ALPV from *V. velutina* and *A. ceranae* are indicated by a black dot and asterisk, respectively (GenBank accession no. MF795134, MF795135). (**A**) Tree branches are colored according to the strains originating from different regions. (**B**) Inferred host states were reconstructed in the MCC tree according to different hosts. (**C**) Phylogenetically inferred migration rates for ALPV. The number indicates the Bayes factor (BF). The picture of the aphid is from Reference [13], and the pictures of *V. velutina* and *A. cerana* were photographed by Chunsheng Hou. The weight of line indicates the Bayes factor support for migration rates (from thin to thick arrows: BF = 3–10, >100).

Phylogenetic analyses using full-length nucleotide sequences of ALPVs from different regions showed that ALPV strains can be divided into four major lineages originating from China, Kenya, USA, and the Netherlands (Figure 1A). ALPV-Vv and ALPV-Ac were most closely associated with those from China, except for two isolates (JQ320375 and KX884276), which were closely related to USA strains. Evidence of cross species migration was indicated through the analysis of ALPV RdRp from different hosts. The reconstructed MCC tree of ALPV hosts showed that ALPV-Ac and ALPV-Vv were closely associated with aphid strains, but totally differed from those of *A. mellifera*, apart from one (MH223645), and suggested that the route of migration of ALPV among these species was from aphids to *V. velutina*, then to *A. cerana* (Figure 1B). Additionally, there was strong support for migration from aphids to *V. velutina* (BF = 207.6), and a more weakly supported route from *V. velutina* to *A. cerana*, the BF being slightly higher than 3 (BF = 4.4) (Figure 1C). 

## 4. Discussion

Our results show ALPV was present in the Chinese *V. velutina*. In fact, Yañez et al. [6] had detected IAPV in *V. velutina* as well as DWV and black queen cell virus (BQCV) in *Vespula vulgaris* [19]. While *V. velutina* and *A. cerana* frequently live in a mountainous areas, the results of Figure 1B,C demonstrate ALPV might have an extended host range from *V. velutina,* a predator of honey bees, to *A. cerana* by increasing the opportunity for contact in the wild [20,21]. More importantly, mixed infections between different hosts could result in the emergence of new viruses through genetic recombination, thus potentially enhancing its virulence and expanding the host range [22]. As shown in Figure 1B, although ALPV strains from *A. mellifera* were significantly different from those of *A. cerana* and *V. velutina*, there was still one from *A. mellifera* that clustered with the aphid group. This demonstrated that ALPV could form a cyclic transmission among *A. mellifera* aphids. Due to the limited ALPV data from *A. mellifera* and *A. cerana*, our results could not tell the potential migration pathway between *A. mellifera* and *A. cerana*. Moreover, we still could not conclude that ALPV in *A. cerana* is from *V. velutina*. Therefore, ALPV was not found in our *A. mellifera* samples, but it does not mean they did not carry ALPV. A recent study suggested that ALPV was first found in *A. mellifera* in Spain, this also being the first reported instance in Europe [23]. The ability of ALPV to jump between host species is feasible given the wide range of insect species that can be infected by this virus around the world. These findings highlight the importance of the concern over the global distribution and prevalence of ALPV in pollinators. 

On the other hand, the presence of pathogens or parasites could limit the spread of *V. velutina* populations [11,19]. For example, *Conops vesicularis* can parasitize the queen of *V. velutina* and lead to the potential reduction of *V. velutina* populations [11,24]. In addition, a mermithid nematode was also found that can parasite the adult *V. velutina* [11]. However, these two studies need to clearly investigate the characteristics of these parasites before using them as biological control agents, such as host specificity. Similarly, despite confirming the presence of ALPV in *V. velutina* and *A. cerana,* we still need to identify the difference in virulence between *V. velutina* and *A. cerana*, although we did find that *V. velutina* was more susceptible to ALPV than *A. cerana* according to our analysis on viral replication ability. Therefore, the current study provides an important first step in understanding this viral infection and may potentially lead to a way to control *V. velutina* by employing the virus. 

In honey bees, the arrival of a parasitic disease vector, *V. destructor*, generated a chance for sharply enhanced virus transmission, which may have induced a shift towards increased virulence in viruses such as DWV and IAPV that already existed in honey bee populations, but had previously persisted as asymptomatic infections at a low level [7,25]. However, whether emerging viral diversity itself could be a major driving force of honey bee colony losses, as may be the case for ALPV in the current study, cannot yet be determined. Together with the results presented herein, these analyses indicate that the role of hornets in the epidemiology and emergence of pollinator viruses needs further investigation to assess the potential prevalence, virulence, and hazards of ALPV in managed bee colonies around the world, as well as other vectors such as *V. destructor* and the small hive beetle [26].

## 5. Conclusions

The current study aimed to ascertain whether ALPV was present in *V. velutina* and *A. cerana* samples from China. Our results showed that *V. velutina* and *A. cerana* had been infected with ALPV. Furthermore, we found that ALPV-Ac and ALPV-Vv were closely associated with aphid strains using a phylogenetic method. This study indicated that ALPV had expanded host range, but the exact route of migration of ALPV between *A. mellifera*, *A. cerana*, and *V. velutina* needs to be further clarified.

## Supplementary Materials

The following are available online at https://www.mdpi.com/2075-4450/10/6/157/s1, Figure S1: Identified the conserved motif in dicistroviruses and confirmed the replication of ALPV in *V. velutina* and *A. cerana*. (a) Hornets caught the honey bees in front of hive. (b) Detection the ALPV in *V. velutina* and *A. cerana*. Lane M: DNA ladder. Lane 1: *A. mellifera* samples from where we collected *A. cerana*. Lane 2: *A. cerana* samples. Lane 3: *A. mellifera* samples from where we collected *V. velutina*. Lane 4: *V. velutina* samples. (c) ALPV replication in *A. cerana* and *V. velutina*. (d) Highly conserved sequences identified in dicistroviruses. The number of copies of positive and negative RNA strands was evaluated using absolute quantification by quantitative PCR. Table S1: Primers used for detected and amplified the ALPV in current study.
