# Supplementary material for: Discovery of Aphid Lethal Paralysis Virus in Vespa velutina and Apis cerana in China
Source: Insects. 2019 Jun 3;10(6):157. doi: 10.3390/insects10060157 (PMC6628042; doi:10.3390/insects10060157)
Supplement: Supplementary file 1 [file insects-10-00157-s001.pdf]

Supplementary Materials

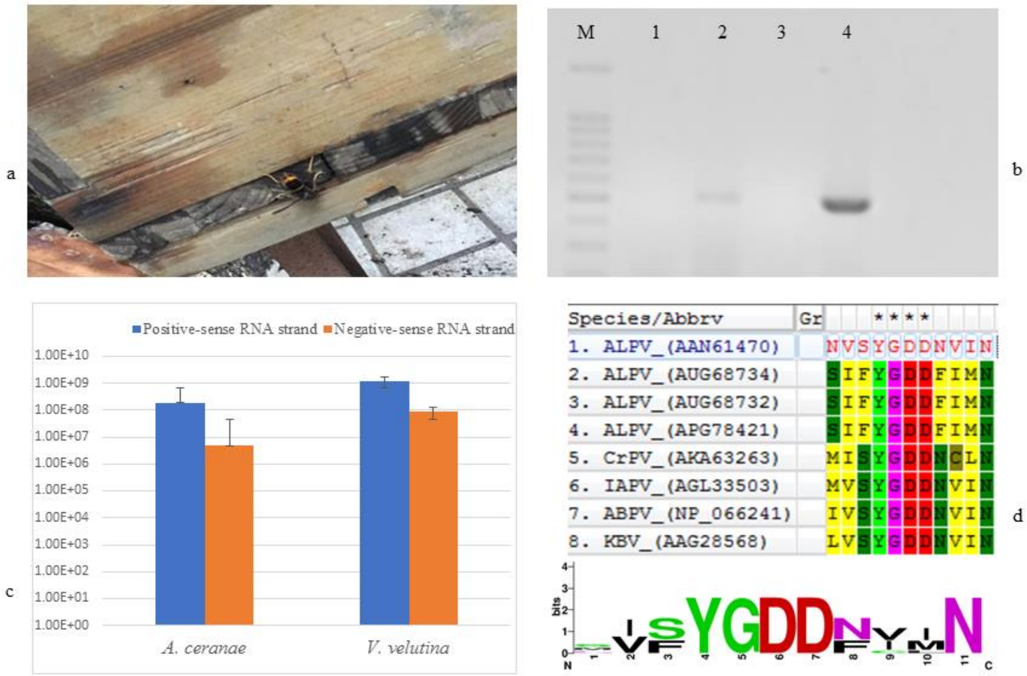

Figure S1. Identified the conserved motif in dicistroviruses and confirmed the replication of ALPV in V. velutina and A. cerana. (a) Hornets caught the honey bees in front of hive. (b) Detection the ALPV in V. velutina and A. cerana. Lane M: DNA ladder. Lane 1: A. mellifera samples from where we collected A. cerana. Lane 2: A. cerana samples. Lane 3: A. mellifera samples from where we collected V. velutina. Lane 4: V. velutina samples. (c) ALPV replication in A. cerana and V. velutina. (d) Highly conserved sequences identified in dicistroviruses. The number of copies of positive and negative RNA strands was evaluated using absolute quantification by quantitative PCR.

Table S1. Primers used for detected and amplified the ALPV in current study.

Position	Sequence (5'-3')	Reference
1F	TTAAATAAGAAACTATATATTCTTACAATATACATTT	This study
1937R	CAGTATTATTAAATCGGTTGTGTTT	This study
1847F	TTCCCGAAAGCAATTTCTGG	This study
4346R	GGAAGTGTTTCGGTATCATACCT	This study
3875F	CGACTAATCGACTCACACGCT	This study
6800R	ATCAAAATAGGACATTCGGACTAAT	This study
6183F	TCATAATGAATATACGACCAGAGGT	This study
8894R	CCGCGCCGCGTAAAC	This study
8843F	TTATCTATGCCACGACAACCGAAGG	This study
9835R	AGAAAAATAACATTTACATACAATACGTAC	This study
ALPV F	GCGTACCATACTACTCACCATATTTATTA	Dombrovsky et al., 2013
ALPV R	AGTTAATCCATAAAGTGCAATCTACAATAC	Dombrovsky et al., 2013
TaqF	AGCCTGCGCACCGTGGCTTGGCGGAGATTTTGAG	This study
TaqR	AGCCTGCGCACCGTGGTGACCTATCTGACCACCTAATC AAAAT	This study

Reference

Dombrovsky A, Luria N. The *Nerium oleander* aphid *Aphis nerii* is tolerant to a local isolate of *Aphid lethal paralysis virus* (ALPV). Virus Genes. 2013;46:354-361.
